# Association of Continuation and Maintenance Electroconvulsive Therapy With Relapse and Hospitalization Outcomes: A Retrospective Study of 134 Patients

**DOI:** 10.7759/cureus.110650

**Published:** 2026-06-11

**Authors:** Younes El Fatmaoui, Siham Belbachir, Fouad Aziz

**Affiliations:** 1 Ar-Razi Psychiatric University Hospital, Centre Hospitalier Ibn Sina, Salé, MAR; 2 Psychiatry, Grand Hôpital de l'Est Francilien Site de Marne-la-Vallée, Jossigny, FRA

**Keywords:** continuation electroconvulsive therapy, electroconvulsive therapy, maintenance electroconvulsive therapy, mood disorders, psychiatric hospitalization, psychotic disorders, relapse prevention

## Abstract

Introduction: Electroconvulsive therapy is an effective treatment for severe mood and psychotic disorders. However, remission after an acute course does not always prevent subsequent relapse. Continuation and maintenance electroconvulsive therapy may be used after the index course to reduce relapse risk and psychiatric hospitalization. The primary objective of this study was to examine the association between continuation or maintenance electroconvulsive therapy and relapse outcomes after an acute electroconvulsive therapy course. Secondary exploratory objectives were to assess hospitalization outcomes, diagnostic subgroup differences, and electroconvulsive therapy-related clinical characteristics.

Methods: We conducted a retrospective observational study including 134 patients who received electroconvulsive therapy in the psychiatric unit of the Grand Hôpital de l’Est Francilien, France, between January 2015 and January 2025. Sociodemographic and clinical characteristics, diagnosis, indication for electroconvulsive therapy, acute treatment parameters, use and duration of continuation or maintenance electroconvulsive therapy, Brief Psychiatric Rating Scale scores, adverse effects, relapse, rehospitalization, and length of hospital stay were collected from medical records. Patients receiving continuation or maintenance electroconvulsive therapy were compared according to treatment duration, with a distinction between protocols lasting six months or less and those continued beyond six months.

Results: Among the 134 included patients, 53 patients received continuation or maintenance electroconvulsive therapy, and 81 did not. The mean age was 49.46 years, and 61.2% of patients were female. The most frequent diagnoses were unipolar depression, bipolar disorder, schizophrenia, and schizoaffective disorder. The acute course was associated with a substantial clinical improvement, with mean Brief Psychiatric Rating Scale scores decreasing from 69.6 before electroconvulsive therapy to 30.9 after treatment. Among patients receiving continuation or maintenance electroconvulsive therapy, those treated for more than six months had significantly fewer late relapses than those treated for six months or less. Electroconvulsive therapy was also associated with a significant reduction in hospital length of stay among patients with early relapse. Patients with psychotic disorders had higher relapse rates than those with mood disorders, particularly beyond six months.

Conclusions: In this retrospective cohort, the strongest signal was observed within the continuation/maintenance electroconvulsive therapy subgroup, where treatment beyond six months was associated with fewer late relapses than treatment lasting six months or less. Because of the retrospective non-randomized design, these findings should be interpreted as associative and hypothesis-generating rather than causal and support the integration of individualized maintenance electroconvulsive therapy strategies after an acute course while highlighting the need for prospective studies to define optimal duration and frequency.

## Introduction

Electroconvulsive therapy is a well-established treatment for severe psychiatric disorders, particularly mood disorders and psychotic disorders. It is commonly used in patients with severe depressive episodes, bipolar disorder, schizophrenia, schizoaffective disorder, catatonia, acute suicidal risk, or treatment-resistant symptoms [1-7]. Although an acute course of electroconvulsive therapy can lead to substantial clinical improvement or remission, it does not necessarily prevent subsequent relapse [1-3,7].

Relapse after a successful acute course of electroconvulsive therapy remains a major clinical concern. Many patients continue to present a high risk of symptom recurrence, rehospitalization, and functional deterioration, especially when they have severe, recurrent, or treatment-resistant psychiatric disorders [1-3,7-9]. Therefore, relapse prevention strategies should be considered after the index electroconvulsive therapy course, particularly in patients with a history of rapid relapse, severe symptoms, psychotic features, or poor tolerance or insufficient response to pharmacological treatment [1-7].

Continuation electroconvulsive therapy refers to electroconvulsive therapy sessions administered during the months following the acute course, generally within the first six months after clinical response or remission. Maintenance electroconvulsive therapy is usually defined as a more prolonged treatment strategy, with sessions administered at wider intervals over a longer period in order to sustain clinical stability and reduce the risk of relapse [1-3,7]. In clinical practice, continuation and maintenance electroconvulsive therapy protocols vary widely in terms of duration, frequency, and patient selection [2,3,6,7].

Despite increasing clinical use, the association of continuation and maintenance electroconvulsive therapy with relapse and psychiatric hospitalization outcomes remains an important area of investigation [1-3,6-9]. In particular, real-world data are needed regarding its relationship with early and late relapse, length of hospital stay, and rehospitalization rates across heterogeneous psychiatric populations [8,9].

The primary objective of this study was to examine the association between continuation or maintenance electroconvulsive therapy and relapse outcomes after an acute electroconvulsive therapy course. Secondary exploratory objectives were to assess hospitalization outcomes, diagnostic subgroup differences, and electroconvulsive therapy-related clinical characteristics.

## Materials and methods

Study design and setting

This retrospective observational study was conducted using the medical records of all patients who received electroconvulsive therapy in the psychiatric unit of the Grand Hôpital de l’Est Francilien, France, between January 2015 and January 2025. The study included patients who underwent an acute course of electroconvulsive therapy and, when applicable, continuation or maintenance electroconvulsive therapy.

Because of its retrospective design and the use of existing medical records, the study was exempted from formal ethics committee approval. All data were collected and analyzed anonymously, and no identifying patient information was included in the manuscript.

Inclusion and exclusion criteria

Patients were included if their medical records contained sufficient clinical and hospitalization data for analysis. Patients were eligible regardless of whether they subsequently received continuation or maintenance electroconvulsive therapy.

Patients were excluded if electroconvulsive therapy was performed outside the study period, if the available medical record did not allow extraction of the main study outcomes, or if follow-up information regarding relapse or rehospitalization was unavailable. 

Patients with missing data for a specific variable were excluded only from the analysis involving that variable. No imputation of missing data was performed.

Outcome definitions

Relapse was defined as clinical worsening documented in the medical record and/or leading to psychiatric rehospitalization after the index acute electroconvulsive therapy course. Early relapse was defined as a relapse occurring within six months after the index acute electroconvulsive therapy course. Late relapse was defined as a relapse occurring beyond six months after the index acute electroconvulsive therapy course.

Rehospitalization was defined as a new psychiatric inpatient admission occurring after the index electroconvulsive therapy course. Length of hospital stay was defined as the number of inpatient days related to hospitalization for relapse. The annual rehospitalization rate was defined as the number of psychiatric rehospitalizations per year before and after electroconvulsive therapy.

Early and late relapse categories were analyzed separately according to the timing of relapse after the index acute electroconvulsive therapy course. 

Electroconvulsive therapy procedure and treatment characteristics

Before electroconvulsive therapy, all patients underwent psychiatric and anesthetic assessments. Electroconvulsive therapy was performed in the post-anesthesia care unit by a multidisciplinary team including a psychiatrist, an anesthesiologist, psychiatric nurses, and anesthesia nurses. Electroconvulsive therapy was administered under general anesthesia induced with propofol and muscle relaxation with succinylcholine.

Treatments were delivered using brief-pulse stimulation with a Thymatron System IV device (Somatics, LLC, Venice, FL, USA). Electrode placement was determined according to clinical judgment and was classified as bitemporal or right unilateral stimulation. Acute electroconvulsive therapy frequency was categorized as one, two, or three sessions per week. The total number of acute electroconvulsive therapy sessions was extracted from medical records.

Continuation and maintenance electroconvulsive therapy characteristics were also collected, including whether continuation or maintenance electroconvulsive therapy was performed, treatment duration, treatment rhythm, and frequency of sessions. Continuation and maintenance protocols were categorized according to duration as six months or less versus more than six months. The continuation or maintenance rhythm was categorized as monthly or twice monthly.

Concomitant psychotropic treatment was generally continued during and after electroconvulsive therapy. In this cohort, 132 patients (98.5%) continued psychotropic treatment after electroconvulsive therapy, reflecting routine clinical practice in which electroconvulsive therapy was used as part of a broader therapeutic strategy rather than as a stand-alone intervention.

Data collection

Data were extracted from medical records. The collected variables included sociodemographic characteristics, primary psychiatric diagnosis, indication for electroconvulsive therapy, duration of the episode before electroconvulsive therapy indication, ineffective pharmacological treatments before electroconvulsive therapy, total number and frequency of acute electroconvulsive therapy sessions, use of continuation or maintenance electroconvulsive therapy, duration and frequency of continuation or maintenance electroconvulsive therapy, adverse effects, relapse or rehospitalization within six months after electroconvulsive therapy, relapse or rehospitalization beyond six months, length of hospital stay before and after electroconvulsive therapy in patients with early relapse, and annual number of rehospitalizations before and after electroconvulsive therapy in patients with late relapse. Clinical severity was assessed using the Brief Psychiatric Rating Scale scores before and after electroconvulsive therapy [10].

Patients who received continuation or maintenance electroconvulsive therapy were divided into two subgroups according to treatment duration. The continuation or short-duration electroconvulsive therapy subgroup included patients who received continuation or maintenance treatment for six months or less. The prolonged maintenance electroconvulsive therapy subgroup included patients who continued treatment for more than six months.

For subgroup analyses, patients were also classified according to diagnostic category. The psychotic disorder group included patients with schizophrenia or schizoaffective disorder. The mood disorder group included patients with unipolar depression or bipolar disorder.

Statistical analysis

Qualitative variables were described as n (%). Quantitative variables were described as mean ± standard deviation or median with interquartile range, as appropriate. Comparisons between qualitative variables were performed using the chi-square test or Fisher’s exact test, depending on expected cell counts. Paired quantitative variables before and after electroconvulsive therapy were analyzed using the Wilcoxon signed-rank test. Comparisons involving more than two independent groups were performed using the Kruskal-Wallis test when appropriate.

Statistical analyses were performed using Python version 3.13.5 (Python Software Foundation, Fredericksburg, VA, USA) with the SciPy statistical package. A p-value of less than 0.05 was considered statistically significant. No post hoc pairwise comparisons were performed unless otherwise specified. No correction for multiple comparisons was applied; therefore, subgroup analyses were considered exploratory.

## Results

Patient characteristics

A total of 134 patients were included in the study. All patients received an acute course of electroconvulsive therapy. Among them, 53 patients (39.6%) received continuation or maintenance electroconvulsive therapy, whereas 81 patients (60.4%) did not. The cohort was predominantly female, with 82 women (61.2%) and 52 men (38.8%).

The mean age at the time of electroconvulsive therapy was 50.0 ± 16.3 years. The median age was 51.5 years, with an interquartile range of 37.0-64.8 years and a range from 20 to 89 years. Marital status, employment status, and primary psychiatric diagnoses are summarized in Table 1. The most frequent primary diagnoses were unipolar depression in 46 patients (34.3%), bipolar disorder in 20 patients (14.9%), schizophrenia in 26 patients (19.4%), and schizoaffective disorder in 24 patients (17.9%). Other diagnoses were less frequent and included obsessive-compulsive disorder, postpartum depression, and epilepsy, each reported in one patient (0.7%). The demographic and clinical characteristics of the study population are summarized in Table 1.

**Table 1:** Demographic and clinical characteristics of the study population. Data are presented as n (%) unless otherwise specified. Age is presented as mean, median, and range. ECT: electroconvulsive therapy; DSM-5: Diagnostic and Statistical Manual of Mental Disorders, Fifth Edition [11].

Variable	Results
Number of patients	N = 134
Age at the time of ECT	Mean ± SD: 50.0 ± 16.3 years; median (IQR): 51.5 (37.0–64.8); range: 20–89 years
Sex	52 (38.8%) men / 82 (61.2%) women
Marital status	
Single	56 (41.8%)
Married	37 (27.6%)
Divorced	28 (20.9%)
Widowed	13 (9.7%)
Employment status	
Employed	23 (17.2%)
Student	6 (4.5%)
Unemployed	69 (51.5%)
Retired	36 (26.9%)
Primary diagnosis (DSM-5)	
Unipolar depression	46 (34.3%)
Bipolar depression	20 (14.9%)
Bipolar mania	15 (11.2%)
Schizophrenia	26 (19.4%)
Schizoaffective disorder	24 (17.9%)
Obsessive-compulsive disorder	1 (0.7%)
Postpartum depression	1 (0.7%)
Epilepsy	1 (0.7%)

Indications for electroconvulsive therapy

The mean duration of the psychiatric episode before the indication for electroconvulsive therapy was 99.6 ± 112.9 days, with a range from two to 730 days. This duration differed significantly according to the indication for electroconvulsive therapy (p = 0.0049).

The most frequent indication was resistance to pharmacological treatment, observed in 56 patients (41.8%). This indication was associated with the longest mean episode duration before electroconvulsive therapy, at 248.5 ± 179.4 days. Severe psychotic features were reported in 34 patients (25.4%) and acute suicidal risk in 37 patients (27.6%), with shorter mean durations before electroconvulsive therapy of 45.7 ± 41.3 days and 34.2 ± 26.7 days, respectively. Catatonic features were observed in seven patients (5.2%) and were associated with a mean duration of 28.4 ± 18.7 days before electroconvulsive therapy.

The duration of the psychiatric episode before electroconvulsive therapy varied significantly according to the clinical indication, as shown in Table 2.

**Table 2:** Duration of the psychiatric episode before electroconvulsive therapy according to clinical indication. Data are presented as n (%) and mean ± SD. Statistical comparison was performed using the Kruskal-Wallis test or ANOVA, according to data distribution. A p-value < 0.05 was considered statistically significant. ECT: electroconvulsive therapy

Indication for ECT	N	%	Mean duration of the episode before ECT (days)
Resistance to pharmacological treatment	56	41.8%	248.5 ± 179.4
Severe psychotic features	34	25.4%	45.7 ± 41.3
Acute suicidal state	37	27.6%	34.2 ± 26.7
Catatonic features	7	5.2%	28.4 ± 18.7

Among patients with pharmacological treatment resistance, the mean number of psychotropic medications tried before electroconvulsive therapy was 2.9 per patient. The most frequently unsuccessful medications were quetiapine (42.6%), aripiprazole (35.2%), olanzapine (31.5%), and venlafaxine (29.6%). Other treatments included risperidone, amisulpride, valproate, lithium, mianserin, duloxetine, paroxetine, fluoxetine, escitalopram, vortioxetine, clomipramine, amitriptyline, and clozapine.

**Figure 1:**
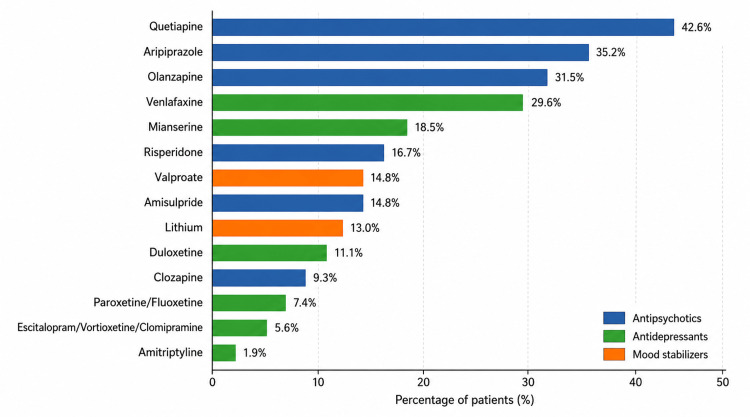
Unsuccessful pharmacological treatments before electroconvulsive therapy among patients with pharmacological treatment resistance (n = 56). Percentages represent the proportion of patients previously exposed unsuccessfully to each medication. Patients may have received more than one medication; therefore, percentages do not sum to 100%.

The unsuccessful pharmacological strategies before electroconvulsive therapy are presented in Figure 1.

Acute electroconvulsive therapy course

During the acute phase, patients received a mean of 12.18 ± 12 electroconvulsive therapy sessions. A 12-session course was the most frequent protocol, representing approximately 42.5% of patients. The minimum number of sessions was three, and the maximum was 32.

Bitemporal stimulation was used in 123 patients (91.8%), whereas right unilateral stimulation was used in 11 patients (8.2%). Most patients received two sessions per week (n = 84, 62.7%), while 44 patients (32.8%) received one session per week and six patients (4.5%) received three sessions per week.

Continuation and maintenance of electroconvulsive therapy

Among the 53 patients (39.6%) who received continuation or maintenance electroconvulsive therapy, the duration of treatment ranged from one to 24 months. Twenty patients (37.7%) received continuation electroconvulsive therapy for less than six months, 16 patients (30.2%) received treatment for exactly six months, and 17 patients (32.1%) received maintenance electroconvulsive therapy for more than six months.

The most frequent continuation or maintenance schedule was monthly, used in 34 patients (64.2%), while 19 patients (35.8%) received sessions twice monthly.

Clinical response

After the acute course of electroconvulsive therapy, complete clinical improvement was observed in 80 patients (59.7%), while partial improvement was observed in 42 patients (31.3%). Ten patients (7.5%) showed no clinically significant improvement, and two medical records (1.5%) were not evaluable for this variable.

The mean Brief Psychiatric Rating Scale score decreased from 69.6 ± 17.8 before electroconvulsive therapy to 30.9 ± 12.5 after treatment. This corresponded to a mean reduction of 38.7 points and an improvement of more than 55% in symptom severity.

The number of sessions required before clinical improvement differed according to diagnosis. Patients treated for catatonia showed the earliest improvement, after a mean of 4.7 ± 2.1 sessions. Patients with schizophrenia and schizoaffective disorder required a higher number of sessions before improvement, with means of 9.8 and 9.4 sessions, respectively. This difference was statistically significant (p = 0.0049).

**Figure 2:**
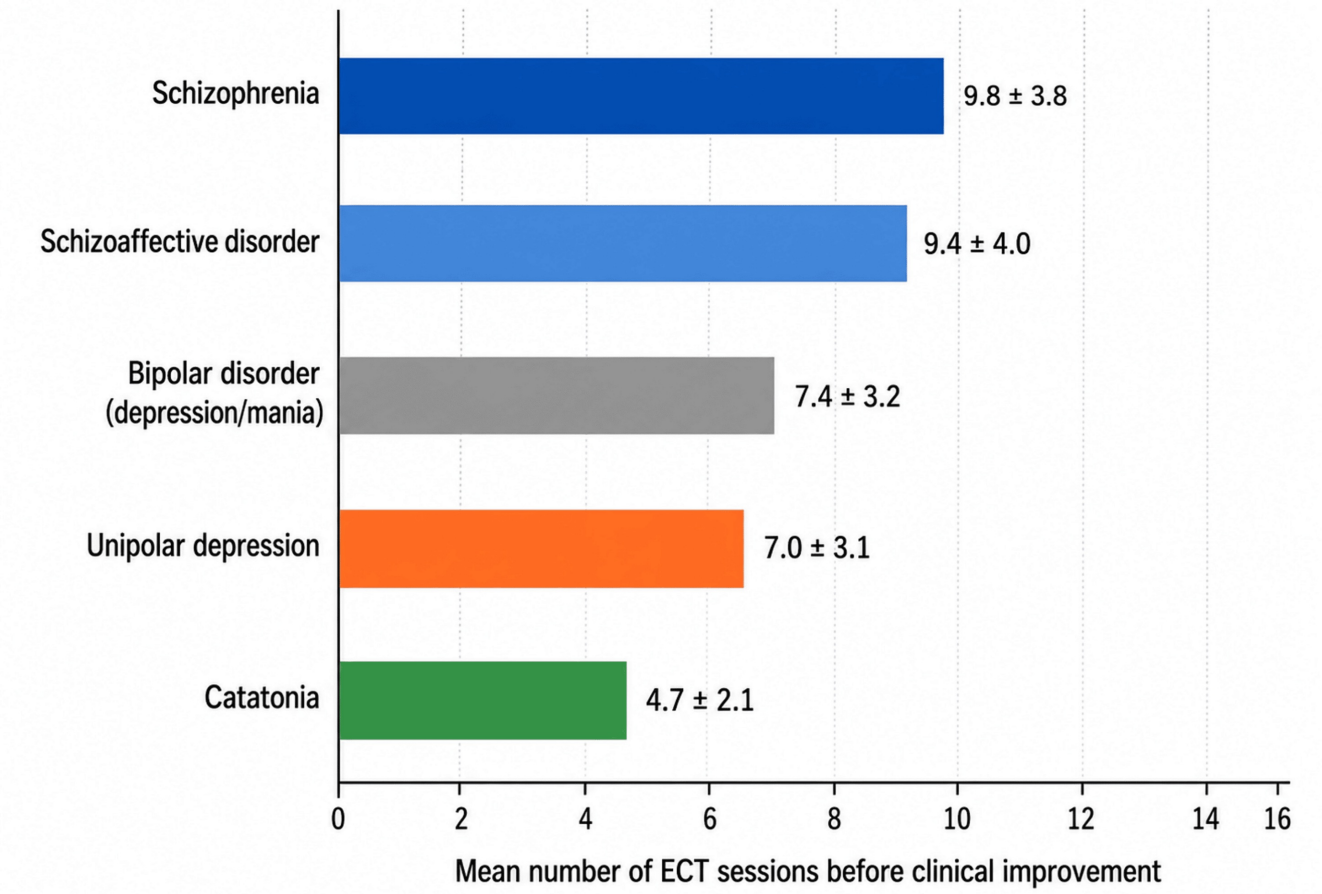
Mean number of electroconvulsive therapy sessions required before clinical improvement according to diagnosis. Bipolar depression and bipolar mania were combined into a single bipolar disorder category for this analysis. Data are presented as mean ± standard deviation: catatonia, 4.7 ± 2.1; unipolar depression, 7.0 ± 3.1; bipolar disorder, 7.4 ± 3.2; schizophrenia, 9.8 ± 3.8; and schizoaffective disorder, 9.4 ± 4.0. Group comparison was performed using the Kruskal-Wallis test; p = 0.0049. ECT: electroconvulsive therapy

The mean number of electroconvulsive therapy sessions required before clinical improvement according to diagnosis is shown in Figure 2.

Adverse effects and treatment discontinuation

Adverse effects were reported in 35 patients (26.1%). Cognitive adverse effects were absent in 112 patients (83.6%). Memory impairment was reported in 14 patients (10.4%), concentration or attention difficulties in seven patients (5.2%), confusion in seven patients (5.2%), and disorientation in one patient (0.7%).

Somatic adverse effects were absent in 114 patients (85.07%). Headache was the most frequent somatic adverse effect, reported in 18 patients (13.4%). Vomiting and temporal skin burn were each reported in one patient (0.7%).

Premature discontinuation of electroconvulsive therapy occurred in 20 patients (14.9%). The main reasons were patient refusal, adverse effects, COVID-19 infection, lack of improvement related to very brief seizures, and deterioration of general condition. Adverse effects and reasons for premature discontinuation are summarized in Table 3.

**Table 3:** Adverse effects and reasons for premature discontinuation of electroconvulsive therapy. Data are presented as n (%).

Effects	N (%)
Cognitive effects:	
None	112 (83.6)
Memory impairment	14 (10.4)
Concentration/attention impairment	7 (5.2)
Confusion	7 (5.2)
Disorientation	1 (0.7)
Somatic effects:	
None	114 (85.1)
Headache	18 (13.4)
Vomiting	1 (0.7)
Burn at the temple area	1 (0.7)
Premature discontinuation	20 (14.9)
Reason for discontinuation:	
Patient refusal	9 (6.7)
Side effects	6 (4.5)
COVID-19 infection	4 (2.9)
No improvement (very brief seizures)	1 (0.7)
Deterioration of general condition	1 (0.7)

Relapse and hospitalization outcomes

Among patients who received continuation or maintenance electroconvulsive therapy, 11 patients (20.7%) relapsed within six months. In these patients, the mean length of hospital stay decreased from 104.7 ± 84.3 days before the index electroconvulsive therapy course to 27.2 ± 12.9 days after treatment, corresponding to a 74.0% reduction. This reduction was statistically significant using the Wilcoxon signed-rank test (W = 1.00, p = 0.002).

In the group without continuation or maintenance electroconvulsive therapy, 15 patients (18.5%) relapsed within six months. The mean length of hospital stay decreased from 108.3 ± 82.3 days before electroconvulsive therapy to 29.5 ± 26.9 days after treatment, corresponding to a 72.8% reduction. This reduction was statistically significant using the Wilcoxon signed-rank test (W = 2.00, p = 0.001). The impact of electroconvulsive therapy on the length of hospital stay in patients with early relapse is shown in Table 4.

**Table 4:** Impact of electroconvulsive therapy on length of hospital stay in patients with early relapse. Data are presented as mean ± standard deviation. Paired comparisons were performed using the Wilcoxon signed-rank test. Statistical significance was set at p < 0.05. For the continuation/maintenance electroconvulsive therapy group, W = 1.00 and p = 0.002. For the group without continuation/maintenance electroconvulsive therapy, W = 2.00 and p = 0.001. ECT: electroconvulsive therapy

Group	Mean before ± SD	Mean after ± SD	Reduction %	Wilcoxon signed-rank test	p-value
Maintenance ECT (n = 11, 20.7%)	104.7 ± 84.3 days	27.2 ± 12.9 days	−74.0	W = 1.00	0.002
Without maintenance ECT (n = 15, 18.5%)	108.3 ± 82.3 days	29.5 ± 26.9 days	−72.8	W = 2.00	0.001

Late relapse beyond six months occurred in 27 patients (50.9%) in the continuation or maintenance electroconvulsive therapy group. The annual rehospitalization rate decreased from 1.81 ± 1.08 to 1.70 ± 1.10 admissions per year, but this change was not statistically significant using the Wilcoxon signed-rank test (W = 32.00, p = 0.571).

Late relapse beyond six months occurred in 19 patients (23.5%) in the group without continuation or maintenance electroconvulsive therapy. The annual rehospitalization rate decreased from 1.84 ± 0.96 to 1.58 ± 0.77 admissions per year, without reaching statistical significance using the Wilcoxon signed-rank test (W = 12.50, p = 0.212). Changes in annual rehospitalization rates among patients with late relapse are shown in Table 5.

**Table 5:** Changes in annual rehospitalization rates among patients with late relapse. Data are presented as mean ± standard deviation. Paired comparisons were performed using the Wilcoxon signed-rank test. Statistical significance was set at p < 0.05. For the continuation/maintenance electroconvulsive therapy group, W = 32.00 and p = 0.571. For the group without continuation/maintenance electroconvulsive therapy, W = 12.50 and p = 0.212. ECT: electroconvulsive therapy

Group	Mean before ± SD	Mean after ± SD	Change %	Wilcoxon signed-rank test	p-value
Maintenance ECT (n = 27, 50.9%)	1.81 ± 1.08	1.70 ± 1.10	−6.1	W = 32.00	0.571
Without maintenance ECT (n = 19, 23.5%)	1.84 ± 0.96	1.58 ± 0.77	−14.1	W = 12.50	0.212

Diagnostic subgroup analysis

For subgroup analyses, patients were classified into two diagnostic categories: psychotic disorders, including schizophrenia and schizoaffective disorder, and mood disorders, including unipolar depression and bipolar disorder. The psychotic disorder group included 51 patients (38.1%), while the mood disorder group included 83 patients (61.9%).

Continuation or maintenance electroconvulsive therapy was more frequently used in patients with psychotic disorders than in those with mood disorders, although this difference was not statistically significant using Fisher’s exact test (49.0% vs. 33.7%; odds ratio = 1.89, p = 0.102).

**Figure 3:**
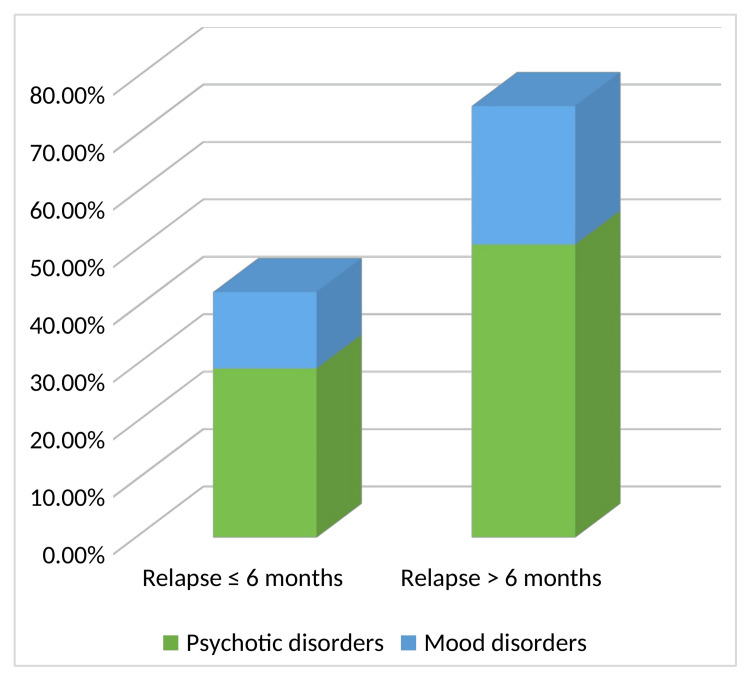
Relapse rates according to diagnostic category. Data are presented as n (%). Group comparisons were performed using Fisher’s exact test. Statistical significance was set at p < 0.05. For early relapse, the odds ratio = 2.73 and p = 0.026. For late relapse, the odds ratio = 3.28 and p = 0.003.

Patients with psychotic disorders had higher relapse rates than those with mood disorders, both within six months (29.4% vs. 13.3%; odds ratio = 2.73, p = 0.026) and beyond six months (51.0% vs. 24.1%; odds ratio = 3.28, p = 0.003), using Fisher’s exact test. Relapse rates according to diagnostic category are presented in Figure 3.

**Figure 4:**
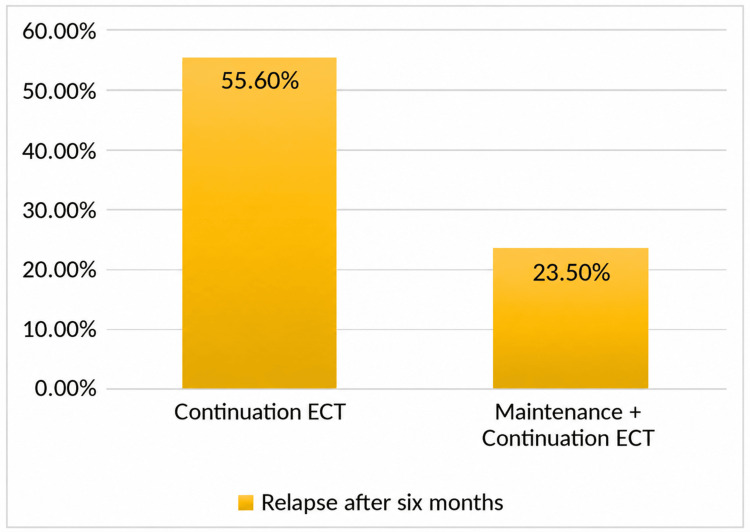
Late relapse rates according to the duration of continuation or maintenance electroconvulsive therapy. Fisher’s exact test: odds ratio = 4.06, p = 0.040. Data are presented as n (%). Group comparison was performed using Fisher’s exact test. Statistical significance was set at p < 0.05. Fisher’s exact test showed odds ratio = 4.06 and p = 0.040. ECT: electroconvulsive therapy

Among patients who received continuation or maintenance electroconvulsive therapy, those treated for six months or less had significantly higher late relapse rates than those who received maintenance electroconvulsive therapy for more than six months (55.6% vs. 23.5%). This difference was statistically significant using Fisher’s exact test (odds ratio = 4.06, p = 0.040). Late relapse rates according to the duration of continuation or maintenance of electroconvulsive therapy are shown in Figure 4.

After the introduction of continuation or maintenance electroconvulsive therapy, total hospital stay decreased significantly in both diagnostic groups. In patients with psychotic disorders, the mean total hospital stay decreased from 145.0 ± 88.8 days before electroconvulsive therapy to 34.9 ± 24.7 days after treatment using the Wilcoxon signed-rank test (W = 1.00, p = 0.001). In patients with mood disorders, it decreased from 54.6 ± 19.2 days to 19.8 ± 13.6 days using the Wilcoxon signed-rank test (W = 2.00, p = 0.003).

In late relapses, the change in annual rehospitalization rate was not statistically significant in either subgroup. In patients with psychotic disorders, the annual rehospitalization rate showed a non-significant trend toward reduction using the Wilcoxon signed-rank test (W = 20.00, p = 0.066). In patients with mood disorders, no significant change was observed using the Wilcoxon signed-rank test (W = 14.00, p = 0.557). Hospitalization outcomes according to diagnostic category are summarized in Table 6.

**Table 6:** Impact of continuation and maintenance electroconvulsive therapy on hospitalization outcomes according to diagnosis. Data are presented as mean ± standard deviation. Paired comparisons were performed using the Wilcoxon signed-rank test. Statistical significance was set at p < 0.05. For the total length of hospital stay, W = 1.00 and p = 0.001 in the psychotic disorder group, and W = 2.00 and p = 0.003 in the mood disorder group. For annual hospitalizations, W = 20.00 and p = 0.066 in the psychotic disorder group, and W = 14.00 and p = 0.557 in the mood disorder group. ECT: electroconvulsive therapy

Diagnostic group	Total length of hospital stay before ECT, mean ± SD	Total length of hospital stay after ECT, mean ± SD	Wilcoxon signed-rank test	p-value	Number of hospitalizations/year before ECT, mean ± SD	Number of hospitalizations/year after ECT, mean ± SD	Wilcoxon signed-rank test	p-value
Psychotic disorders	145.0 ± 88.8 days	34.9 ± 24.7 days	W = 1.00	0.001	1.96 ± 1.11/year	1.58 ± 0.86/year	W = 20.00	0.066
Mood disorders	54.6 ± 19.2 days	19.8 ± 13.6 days	W = 2.00	0.003	1.65 ± 0.88/year	1.75 ± 1.12/year	W = 14.00	0.557

## Discussion

Association of continuation and maintenance electroconvulsive therapy with relapse outcomes

Our results suggest that, within the continuation/maintenance electroconvulsive therapy subgroup, patients who received maintenance electroconvulsive therapy beyond six months had fewer late relapses than those who received continuation or maintenance electroconvulsive therapy for six months or less. This observed association is consistent with previous studies suggesting that continuation and maintenance electroconvulsive therapy may contribute to relapse prevention after an acute electroconvulsive therapy course [1-3,7].

Importantly, this finding should not be interpreted as evidence that continuation or maintenance electroconvulsive therapy was globally superior to no continuation or maintenance treatment. In the crude comparison, late relapse occurred more frequently in the continuation/maintenance electroconvulsive therapy group than in the non-maintenance group. Therefore, the strongest signal in this study concerns the comparison within the continuation/maintenance electroconvulsive therapy subgroup, where treatment beyond six months was associated with fewer late relapses than treatment lasting six months or less.

Data from observational and mirror-image studies also suggest that continuation or maintenance electroconvulsive therapy may be associated with relapse and hospitalization outcomes after several months of treatment continuation [8,9]. This supports the hypothesis that treatment duration may be clinically relevant, although this association should be interpreted cautiously because of the retrospective design and potential selection bias.

The comparison between maintenance strategies was limited to relapses occurring beyond six months. This timing issue should be considered when interpreting early relapse outcomes, particularly because some patients may still have been receiving continuation electroconvulsive therapy during the early follow-up period.

Differences according to diagnostic groups

The diagnostic subgroup analysis showed that psychotic disorders, including schizophrenia and schizoaffective disorder, were associated with a higher risk of relapse than mood disorders, both at six months and beyond six months. This finding is consistent with the clinical literature suggesting that patients with schizophrenia and schizoaffective disorder may require prolonged continuation or maintenance strategies after acute electroconvulsive therapy [4-6].

Several studies, including controlled trials and narrative reviews, suggest that continuation or maintenance electroconvulsive therapy, often combined with antipsychotic treatment, may be associated with sustained clinical stability over prolonged periods in patients with schizophrenia [4-6]. Data from case report reviews indicate that this approach may be continued for several years, with generally satisfactory tolerability and no major signal regarding cognitive adverse effects [6].

In our study, adverse effects were recorded in 26.1% of patients. However, because this was a retrospective chart review without systematic neurocognitive testing, cognitive and other adverse effects may have been under-ascertained. Therefore, tolerability findings should be interpreted cautiously.

In mood disorders, randomized studies and meta-analyses have also reported an association between maintenance electroconvulsive therapy and lower relapse risk, although heterogeneity in protocols and follow-up durations may limit the comparability of results [1-3,7]. Our findings are consistent with this literature, suggesting that some patients remain vulnerable to late relapse when electroconvulsive therapy is discontinued early.

Familial clustering was observed in a small number of cases, including siblings with bipolar disorder and twins with schizophrenia. Because these observations were descriptive and not analyzed systematically, they cannot support conclusions regarding genetic determinants of response and should be explored in future studies.

The literature has widely demonstrated a strong heritability of psychotic disorders and bipolar disorders, with high concordance rates among first-degree relatives and monozygotic twins [12-14]. However, our study was not designed to assess genetic or familial predictors of response to electroconvulsive therapy.

In schizophrenia, continuation or maintenance electroconvulsive therapy has been described as a potential strategy in treatment-resistant forms [4-6]. However, studies specifically exploring the relationship between genetic factors and response to electroconvulsive therapy remain scarce.

Although descriptive, these observations suggest the value of integrating familial or genetic variables into future research in order to better understand the determinants of response and relapse prevention under electroconvulsive therapy.

Impact on hospitalization burden

Beyond relapse outcomes, our study found that electroconvulsive therapy was associated with a shorter length of hospital stay among patients who relapsed early after the index electroconvulsive therapy course. These results are consistent with several mirror-image studies that reported a significant reduction in the number of hospitalization days and readmission rates after the implementation of continuation or maintenance electroconvulsive therapy [8,9].

However, the impact on the annual number of rehospitalizations appeared more moderate and sometimes non-significant, which has also been observed in other observational studies [8]. This suggests that maintenance electroconvulsive therapy may be more closely associated with the severity and duration of relapse episodes than with their absolute frequency, particularly when maintenance treatment is not continued in the long term.

Stimulation modality and relapse

Regarding stimulation modality, our study did not find a significant association between the type of electroconvulsive therapy, unilateral versus bitemporal, and the occurrence of relapse, either at six months or beyond. However, the limited number of patients receiving unilateral stimulation prevents firm conclusions regarding electrode placement.

Limitations and clinical implications

The main limitations of this study are its retrospective design, the absence of randomization, and the heterogeneity of indications and electroconvulsive therapy protocols. Nevertheless, the cohort size, subgroup analysis, and explicit consideration of the time factor strengthen the clinical relevance of the results.

Another important limitation is the absence of a detailed baseline comparability table between exposure groups. Because continuation or maintenance electroconvulsive therapy was prescribed according to clinical judgment rather than randomized assignment, patients who received continuation or maintenance treatment may have differed from those who did not in terms of diagnosis, illness severity, treatment resistance, baseline symptom burden, acute electroconvulsive therapy response, concomitant pharmacotherapy, prior hospitalization history, electrode placement, and number of acute sessions. These differences may have influenced relapse and hospitalization outcomes and may have introduced confounding by indication. Therefore, comparisons between patients with and without continuation or maintenance electroconvulsive therapy should be interpreted cautiously and should not be considered evidence of causal superiority.

Overall, our data suggest that prolonged maintenance electroconvulsive therapy may be clinically relevant, particularly in patients with psychotic disorders, but these findings should be interpreted as associative and hypothesis-generating rather than causal. These findings suggest that maintenance electroconvulsive therapy may be considered as part of individualized relapse prevention strategies, taking into account the diagnostic profile, relapse history, treatment resistance, tolerability, and individual risk.

## Conclusions

In this retrospective cohort, prolonged maintenance electroconvulsive therapy beyond six months was associated with fewer late relapses within the continuation/maintenance electroconvulsive therapy subgroup. Acute electroconvulsive therapy was also associated with substantial symptomatic improvement and reduced hospital length of stay among patients with early relapse.

However, because of the retrospective non-randomized design, potential confounding by indication, concomitant pharmacotherapy, lack of detailed baseline comparability between groups, and possible survivor bias, these findings should be interpreted as associative and hypothesis-generating rather than causal. Prospective studies with standardized outcome definitions, baseline group comparisons, and adjusted analyses are needed to better determine which patients may benefit most from prolonged maintenance electroconvulsive therapy.
